# Venoconstrictor responses to activation of bradykinin‐sensitive pericardial afferents involve the region of the hypothalamic paraventricular nucleus

**DOI:** 10.14814/phy2.15221

**Published:** 2022-03-21

**Authors:** Doug Martin, Casey Reihe, Sam Drummer, Kyle Roessler, Shane Boomer, Madeleine Nelson

**Affiliations:** ^1^ Division of Basic Biomedical Sciences University of South Dakota Vermillion South Dakota USA

**Keywords:** blood pressure, heart rate, hypothalamic paraventricular nucleus, mean circulatory filling pressure, venous tone

## Abstract

Veins are important in the control of venous return, cardiac output, and cardiovascular homeostasis. However, the effector systems modulating venous function remain to be fully elucidated. We demonstrated that activation of bradykinin‐sensitive pericardial afferents elicited systemic venoconstriction. The hypothalamic paraventricular nucleus (PVN) is an important site modulating autonomic outflow to the venous compartment. We tested the hypothesis that the PVN region is involved in the venoconstrictor response to pericardial injection of bradykinin. Rats were anesthetized with urethane/alpha chloralose and instrumented for recording arterial pressure, vena caval pressure, and mean circulatory filling pressure (MCFP), an index of venous tone. The rats were fitted with a pericardial catheter and PVN injector guide tubes. Mean arterial pressure (MAP), heart rate (HR), and MCFP responses to pericardial injection of bradykinin (1, 10 µg/kg) were recorded before and after PVN injection of omega conotoxin GVIA (200 ng/200 nl). Pericardial injection of saline produced no systematic effects on MAP, HR, or MCFP. In contrast, pericardial injection of bradykinin was associated with short latency increases in MAP (16 ± 4 to 18 ± 2 mm Hg) and MCFP 0.35 ± 0.19 to 1.01 ± 0.27 mm Hg. Heart rate responses to pericardial BK were highly variable, but HR was significantly increased (15 ± 9 bpm) at the higher BK dose. Conotoxin injection in the PVN region did not affect baseline values for these variables. However, injection of conotoxin into the area of the PVN largely attenuated the pressor (−1 ± 3 to 6 ± 3 mm Hg), MCFP (−0.19 ± 0.07 to 0.20 ± 0.18 mm Hg), and HR (4 ± 14 bpm) responses to pericardial bradykinin injection. We conclude that the PVN region is involved in the venoconstrictor responses to pericardial bradykinin injection.

## Graphical abstract

Venous function is an integral component of blood pressure regulation. Activation of cardiac sympathetic afferents increases blood pressure and venous tone; however, the brain regions involved in this venoconstrictor response are not known. The data presented, herein, suggest that the region of the hypothalamic paraventricular nucleus is involved in cardiac sympathetic afferent‐mediated venoconstriction.

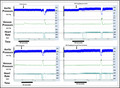

## 1 INTRODUCTION

The venous compartment is important in cardiovascular regulation by virtue of its ability to modulate venous return, cardiac output, and, therefore, arterial blood pressure (Tyberg, 2002). It is recognized that the sympathetic nervous system (SNS) is a major effector system involved in the control of venous tone. Increased venous tone is observed following direct sympathetic nerve stimulation (Bobalova & Mutafova‐Yambolieva, 2001; Kreulen, 2003), reflex activation of the sympathetic nervous system (Pang, 2001), or acute psychological stress (Martin et al., 1996). Moreover, cardiovascular regulatory mechanisms such as the arterial and cardiopulmonary baroreceptors are involved in the control of peripheral venous tone (Pang, 2001; Rothe, 1983). In fact, elegant work by Greene estimated that baroreceptor‐mediated changes in venous tone could alter cardiac output by 40%–60% (Greene & Shoukas, 1986). While, the venous system is an important component of circulatory regulation, outside of the baroreceptor reflexes, the mechanisms that control sympathetic outflow to the venous compartment remain relatively poorly defined.

The heart is endowed with reflexogenic areas that can elicit powerful cardiovascular responses (Chen et al., 2015; Fu & Longhurst, 2009; Hainsworth, 1991). Activation of the cardiac sympathetic afferent reflex (CSAR) increased sympathetic activity, increased heart rate, and arterial vasoconstriction (Hainsworth, 1995; Longhurst et al., 2001; Malliani et al., 1983). While the CSAR has powerful effects on sympathetic outflow (Chen et al., 2015; Xu et al., 2013), until recently, its influence on venous tone was not known. This is of interest because sympathetic control of the arterial and venous compartments may be distinct. Anatomically, it was shown that splanchnic arteries and veins receive different sympathetic nerve projections (Browning et al., 1999). Moreover, functional studies also suggest differential sympathetic control of arteries and veins, at least in the splanchnic region, a major site of venous capacitance (Hottenstein & Kreulen, 1987; Park et al., 2007). Application of bradykinin to sensory afferents in the epicardial layers of the left ventricle can also serve as a useful experimental tool to assess the CSAR (McDermott et al., 1995; Veelken et al., 1996). We recently demonstrated that injection of bradykinin into the pericardial space markedly increased venous tone in the rat (Martin et al., 2020).

In contrast to peripheral sympathetic control of venous function, much less is known about the central nervous system (CNS) sites controlling SNS outflow to the venous compartment. Early studies showed that hypothalamic stimulation constricted veins in the isolated hind limb and intestine (Baum & Hosko, 1965; Cobbold et al., 1964), while mesencephalic stimulation elicited marked constriction of veins in the perfused hind limb (Ueda et al., 1966). Conversely, lesions of the AV3V region of the third ventricle decreased mean circulatory filling pressure (MCFP), an index of integrated venomotor tone, in conscious rats (Bealer, 1993), consistent with tonic CNS control of venous tone. Moreover, our laboratory showed that the disinhibition of the hypothalamic paraventricular nucleus (PVN) resulted in marked increases in venous tone (Martin et al., 1997, 2006) via activation of adrenergic nerves (Martin et al., 2006).

The PVN has been implicated in a wide array of physiologic as well as pathophysiologic cardiovascular responses(Dampney et al., 2018; Patel & Zheng, 2012; Pyner, 2014; Zhou et al., 2019). Current evidence is consistent with a role for the PVN in mediating some of the cardiovascular responses to CSAR activation (Chen et al., 2015). Tract tracing work using pseudorabies virus (PRV) injection into the kidney showed PRV staining in the ventricles and PVN (Gao et al., 2014), consistent with a neuronal connection between these regions. Similarly, activation of the CSAR results in cFos expression in the PVN (Xu et al., 2013). Functional studies are consistent with these anatomical data since manipulation of PVN function can either enhance (Zhu et al., 2004) or attenuate (Xu et al., 2013; Zhong et al., 2008)CSAR evoked blood pressure, heart rate, and/or renal sympathetic nerve responses. Nevertheless, the role of the PVN in mediating CSAR evoked venoconstriction is not known. This work was undertaken to test the hypothesis that the PVN is involved in the venoconstrictor response to pericardial injection of bradykinin.

## 2 MATERIALS AND METHODS

### 2.1 Surgical procedures

Male Sprague Dawley rats (275–400 grams) were purchased from Invigo. The rats were maintained in the Animal Resource Center at the University of South Dakota on a 12‐h day/night cycle and allowed free access to rat chow and tap water. The Institutional Animal Care and Use Committee of the University of South Dakota reviewed and approved all procedures involving these animals and these conformed to the NIH Guide for the Care and Use of Laboratory Animals.

The rats were initially anesthetized with isoflurane, instrumented with a femoral arterial and two femoral venous lines, and then gradually transitioned to anesthesia with intravenous urethane (800 mg/kg) and alpha chloralose (80 mg/kg). A latex‐tipped balloon catheter fashioned from PE‐50 tubing was introduced into the right jugular veins and positioned in the right atrium. The final balloon position was established empirically during surgery as that point where balloon inflation caused cessation of arterial pulsation and a rapid fall of arterial pressure to less than 25 mm Hg. The rats were intubated via a tracheotomy and placed on a ventilator. The left chest was opened at the 4th intercostal space to reveal the thymus gland. The thymus gland was reflected to expose the pericardial sac. A small hole was made in the pericardial sac using a 30‐gauge needle to allow the introduction of an angled PE 10 catheter into the pericardial space. The pericardial catheter was positioned such that the ejection ports lay along the ventrolateral aspects of the left ventricle. The chest was closed in layers, evacuated, and the rats were allowed to breathe spontaneously. The rats were then placed in a Stoelting stereotaxic apparatus for insertion of PVN guide cannulae as we described previously(Martin et al., 2006). Briefly, with the skull level between bregma and lambda, 23‐gauge stainless steel guide cannulae were directed bilaterally at the PVN at a 10° angle from vertical using the following coordinates: 2.0 mm posterior, 1.7 mm lateral to bregma and −6.2 mm ventral from dura. The guide tubes were cemented in place using dental acrylic and the rats were removed from the stereotaxic frame.

### 2.2 Measurement of mean circulatory filling pressure (MCFP)

**FIGURE 1:**
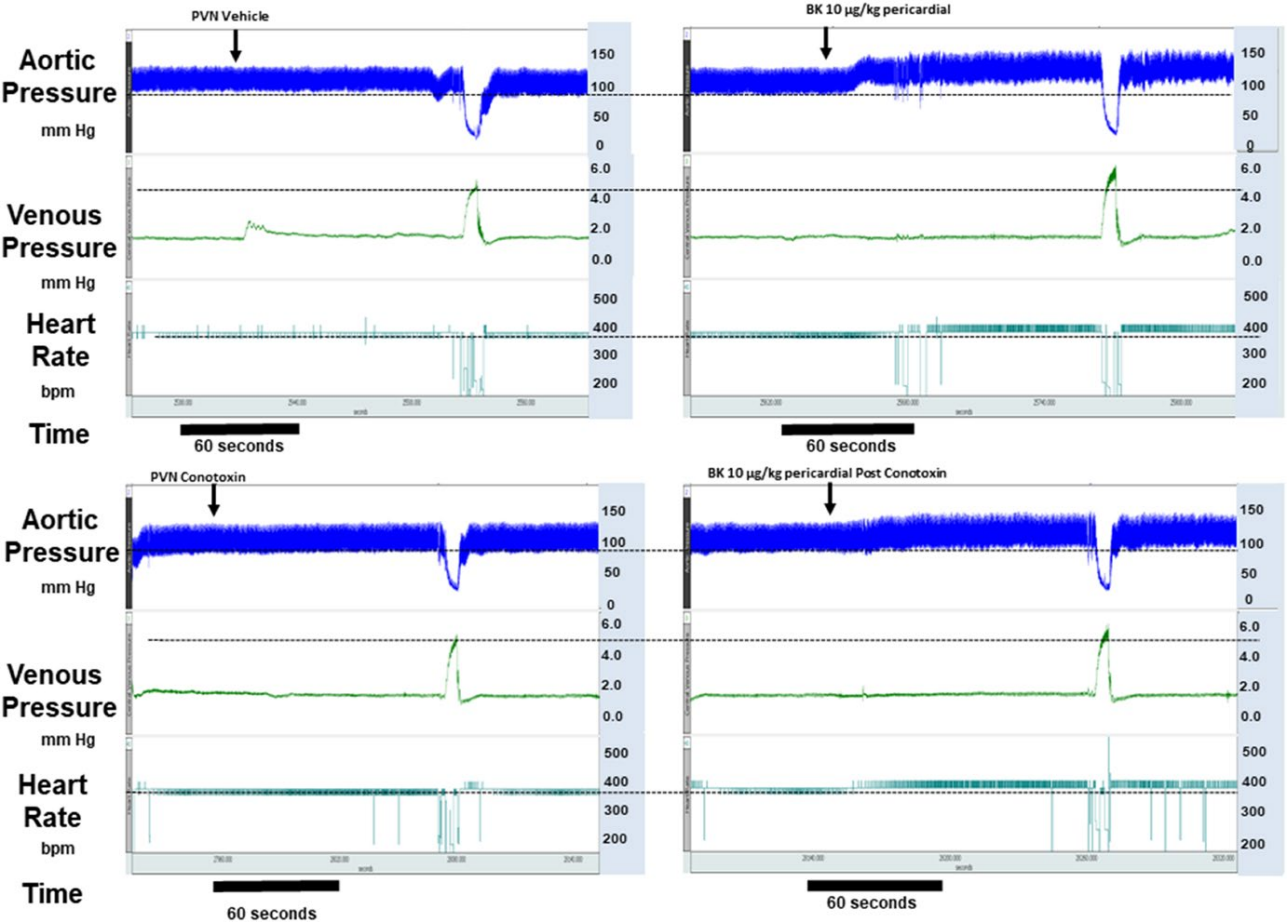
This figure shows tracings for arterial pressure, venous pressure, and heart responses as well as measurement of MCFP during responses to pericardial bradykinin injection before and after pretreatment of the PVN with conotoxin

Mean circulatory filling pressure (MCFP) is an index of integrated venomotor tone (Pang, 1994) that is readily measured in rats during brief interruption of cardiac output. MCFP was calculated from the arterial plateau pressure (AP) and venous plateau pressures recorded after 4 s of right atrial balloon inflation (MCFP = VP + (AP ‐ VP)/60(Martin et al., 2020)) (Figure 1). We found that the time course of the response to pericardial bradykinin was somewhat variable among animals. On occasion, there were short‐lived transients at the onset of the response that then subsided to a more stable level. Others showed a somewhat slower “build” to a sustained level (Figure 1). In all cases, a sustained response was achieved within 90–180 s. Because of the hemodynamic disruption caused by right atrial balloon inflation, we chose to time the right atrial balloon inflations for MCFP determination to occur between 90 and 180 s after BK administration when we felt confident that we measuring a sustained response. Mean values for AP and HR were obtained just prior to right atrial balloon inflation. Arterial blood pressure and heart rate were allowed to return to control levels between successive MCFP measurements.

### 2.3 Experimental protocols

The arterial and venous catheters were connected to pressure transducers, which led to the Biopac data acquisition system. Arterial pressure (AP), heart rate (HR), and venous pressure (VP) were monitored continuously and the animals allowed at least 30–60 min after completion of surgery prior to any interventions. Thereafter, estimates of MCFP were taken at 10–15 min intervals until consistent measurements were obtained.

Mean arterial pressure (MAP), HR, and MCFP responses to pericardial injections of saline or bradykinin (1, 10 µg/kg) (0.1 ml over 5–10 s) were recorded after PVN injection of vehicle or omega conotoxin GVIA (CONO; 200 ng/200 nl per side), an N‐type calcium channel antagonist. We chose to use a relatively large volume of conotoxin injectate based on previous studies using this approach (Sanna et al., 2012) and our desire to inhibit the entire PVN since past experience showed that activation of even a small portion of the PVN might still elicit strong cardiovascular responses. Bilateral PVN injections were accomplished via stainless steel injectors (30 gauge), that extended 1.0 mm beyond the end of the guide cannulae. The injectors were attached to 1.0 µl Hamilton microsyringes (Hamilton Co.) via PE 20 polyethylene tubing. Hemodynamic variables were allowed to stabilize after injector insertion. Two hundred nanoliter injections were then made bilaterally into the PVN over 20 s. In order to serve as both a time and vehicle control, one cohort of rats received two injections of PVN vehicle (Veh/Veh), followed by pericardial BK tested at 10 µg/kg. In the other cohorts, the PVN was injected with vehicle and subsequently with conotoxin. Hemodynamic variables were allowed to stabilize following PVN injection and an atrial balloon inflation was performed. Pericardial injections were then carried out after recovery from the balloon inflation and generally occurred 15–30 min after PVN injection. At the end of the experiments, the PVN injectors were removed and refilled with a solution of bicuculline methiodide (BMI; 1.36 × 10^−3^ M). The injectors were reinserted into the PVN guide tubes and, when recordings had stabilized, 100nl of the BMI solution was injected into the PVN. MAP, HR, and MCFP responses to PVN BMI injection were assessed approximately 2 min following the injection. We used BMI as a test of conotoxin effectiveness based on the following rationale. If conotoxin was effective at inhibiting neurotransmitter release, it should inhibit the release of both GABA and glutamate, which contribute to the mode of action for BMI(Li et al., 2006). On the other hand, if conotoxin was not effective, there should remain some ongoing tonic GABA release the effect of which would be blocked by BMI resulting in the recognized cardiovascular response to PVN BMI administration.

### 2.4 Data analysis

All values are expressed as mean ± SD. Data that met the criteria of normal distribution (Shapiro–Wilk test) and homogeneity of variance (Brown–Forsythe test) were analyzed using parametric statistics. Data that did not meet these criteria (baseline HR, baseline MCFP values, and HR responses) were transformed and the transformed data were analyzed. Analysis of variance for repeated measures was used when comparing the values of MAP, HR, and MCFP within a group. Analysis of variance without repeated measures was used to compare responses among the different cohorts of rats. Post hoc comparisons were performed using the Holm‐Sidak test to correct for multiple comparisons. An unpaired t‐test was used to compare the BMI responses in the absence and presence of PVN conotoxin. Differences were considered significant at *p* < 0.05.

### 2.5 Histology

At the end of the experiments, the animals were euthanized with an overdose of urethane/chloralose anesthetic. Evans blue dye was injected via the pericardial catheter to confirm the location of the pericardial catheter and integrity of the pericardial sac. The rats were then perfused transcardially with 0.9% saline, followed by 4% paraformaldehyde solution and the brains removed. The brains were postfixed in 4% paraformaldehyde for 24 h and then stored in 20% sucrose. Sixty micrometers of frozen coronal sections were cut through the region of the PVN for histological verification of the injection sites. The sections were stained with Cresyl violet. The sites of the injections were determined using Vernier scales on the microscope stage to measure the mediolateral distance from the third ventricle to the mid‐point of the injection tract. The dorsoventral distance was measured from the dorsal aspect of the PVN staining to the point of termination of the injector tract. The anterior‐posterior distance was calculated by adding the number of 60‐µm sections from the first observable PVN staining to the midpoint of the injection tract.

## 3 RESULTS

### 3.1 Control values

Control values for MAP, HR, and MCFP for each cohort obtained before injection of vehicle or conotoxin into the region of the PVN are shown in Table 1. Baseline values for MAP and MCFP were not significantly different in the cohorts destined for time control, BK 1ug, or BK 10ug cohorts. Similarly, there were no statistically significant differences for MAP, HR, or MCFP within each cohort for the four conditions (Table 1). Thus, neither vehicle nor omega conotoxin had an effect on baseline values of MAP, HR, or MCFP.

**TABLE 1:** Baseline values

Vehicle time control			Low‐dose pericardial bradykinin			High‐dose pericardial bradykinin		
(*N* = 5)	Mean	SD	(*N* = 6)	Mean	SD	(*N* = 5)	Mean	SD
Pre PVN Veh control			Pre PVN Veh control			Pre PVN Veh control		
MAP	116	17	MAP	100	16	MAP	111	5
HR	327	40	HR	378	30	HR	375	16
MCFP	5.69	0.73	MCFP	6.49	1.32	MCFP	5.67	1.18
PVN Veh			PVN Veh			PVN Veh		
MAP	113	20	MAP	108	13	MAP	113	6
HR	332	40	HR	373	36	HR	375	19
MCFP	5.95	0.89	MCFP	7.04	1.16	MCFP	5.71	1.19
Pre PVN Veh #2 control			Pre PVN conotoxin control			Pre PVN conotoxin control		
MAP	99	15	MAP	110	11	MAP	108	8
HR	346	42	HR	384	22	HR	383	30
MCFP	5.84	0.74	MCFP	6.76	1.03	MCFP	5.98	1.12
PVN Veh#2			PVN conotoxin			PVN conotoxin		
MAP	102	17	MAP	109	8	MAP	110	7
HR	346	38	HR	387	26	HR	385	30
MCFP	5.85	0.73	MCFP	6.01	1.45	MCFP	5.78	1.18

Note

This table shows the baseline values for mean arterial pressure (MAP), heart rate (HR), and mean circulatory filling pressure (MCFP) in separate cohorts of rats used for the PVN Vehicle Time Control, 1 µg/kg (Low Dose) and 10 µg/kg (High Dose) pericardial bradykinin experiments. Values were obtained before PVN injection of vehicle (Pre PVN Veh Control), after PVN injection of CSF (PVN Veh), before PVN injection of conotoxin (Pre PVN Conotoxin Control) and after PVN injection of conotoxin (200ng/200nl). In the vehicle time control cohort, the PVN was injected with CSF twice (Veh/Veh). Values are mean ± SD. There were no statistically significant differences between the groups for any of the variables.

### 3.2 Pericardial injections

Pericardial injection of saline caused only minor non‐statistically significant changes in blood pressure (−1 ± 1 mm Hg), heart rate (1 ± 3 bpm), and MCFP (−0.15 ± 0.33 mm Hg). In contrast, pericardial injection of bradykinin (BK) was associated with rapid onset changes in blood pressure and venous tone (Figure 1). Pericardial injection of BK increased MAP by 16 ± 4 mm Hg at 1 µg/kg and by 18 ± 2 mm Hg at 10 µg/kg. Similarly, pericardial injection of BK increased MCFP by 0.35 ± 0.19 mm Hg at 1 µg/kg and by 1.01 ± 0.27 mm Hg at 10 µg/kg values that were significantly greater than the response to pericardial saline. In contrast, administration of pericardial BK caused highly variable changes in heart rate, with only the larger dose of bradykinin (10 µg/kg) eliciting a significant increase in heart rate (15 ± 9 bpm) in this cohort.

**FIGURE 2:**
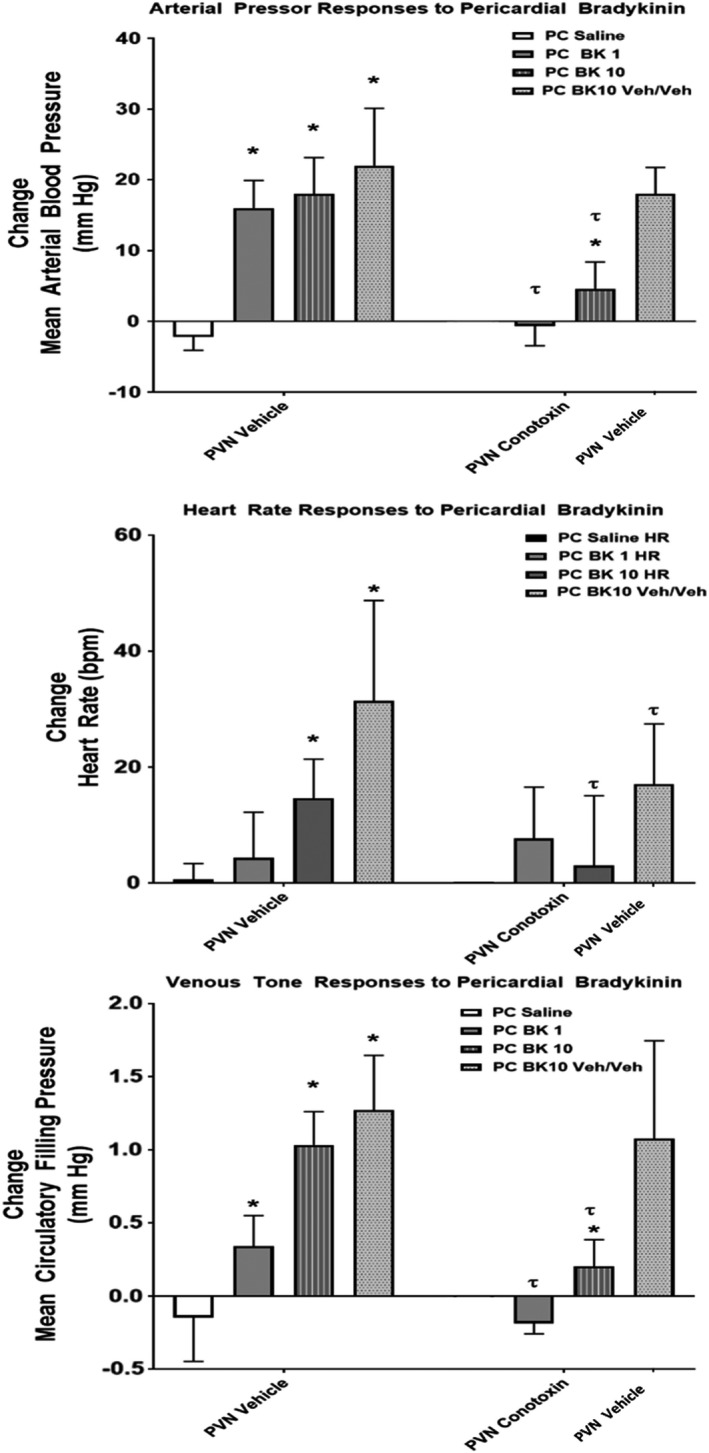
This diagram illustrates changes in mean arterial pressure (MAP), heart rate (HR), and mean circulatory filling pressure (MCFP) caused by pericardial injection of bradykinin (BK) before or after pretreatment of the PVN with omega conotoxin to interrupt neurotransmission or vehicle (Veh/Veh). (n = 6 1 µg/kg; n = 5 10 µg/kg; *n* = 5 veh/veh *p < 0.05 versus pericardial saline; ^#^ *p* < 0.05 pre‐ versus post‐PVN conotoxin; ^τ^ *p* < 0.05 pre‐ versus post‐PVN vehicle (veh/veh controls)

### 3.3 Effects of Injections into the PVN region

**FIGURE 3:**
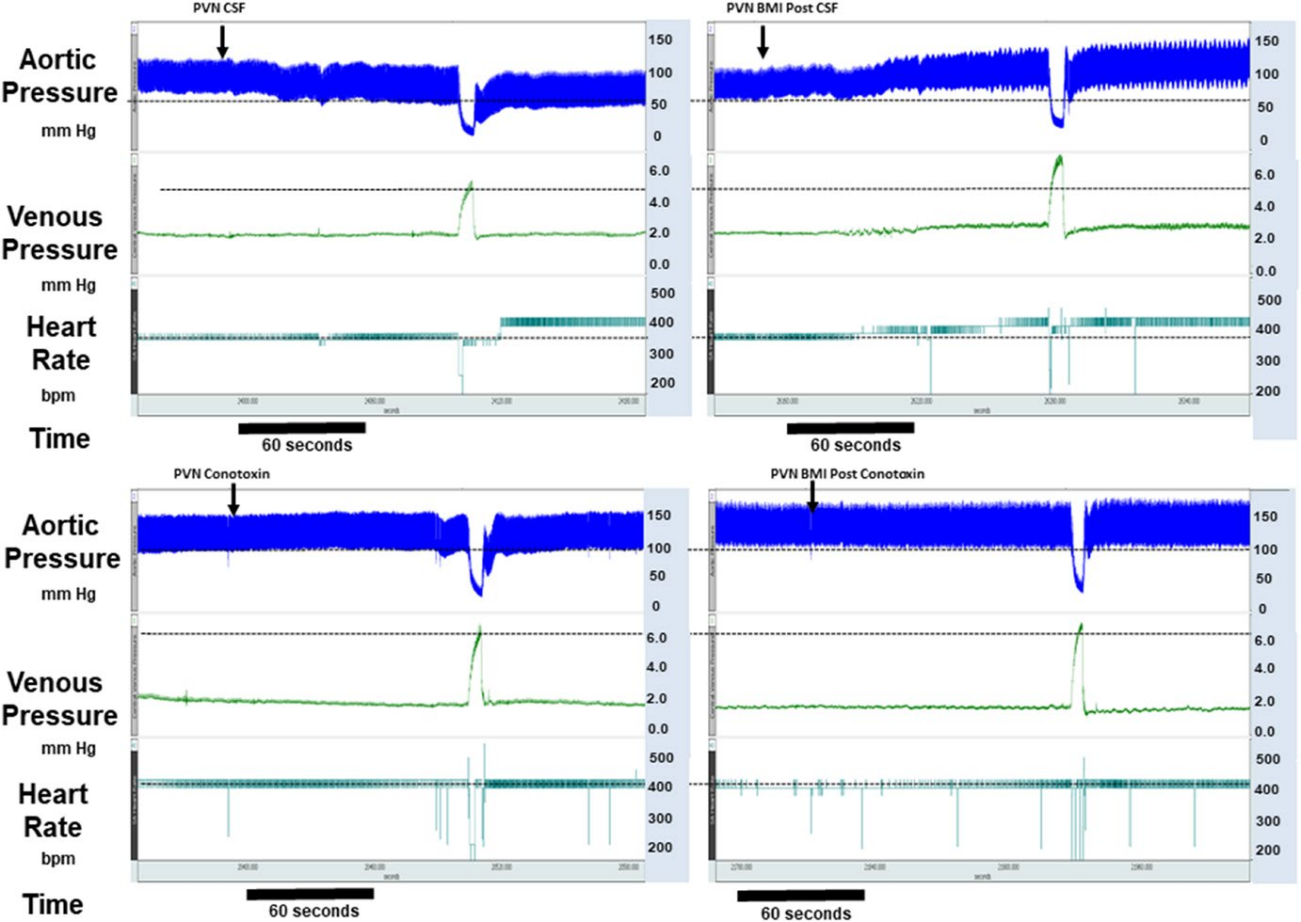
This figure shows tracings for arterial pressure, venous pressure, and heart responses as well as measurement of MCFP during responses to PVN injection of bicuculline (BMI) after PVN pretreatment with vehicle or conotoxin

**FIGURE 4:**
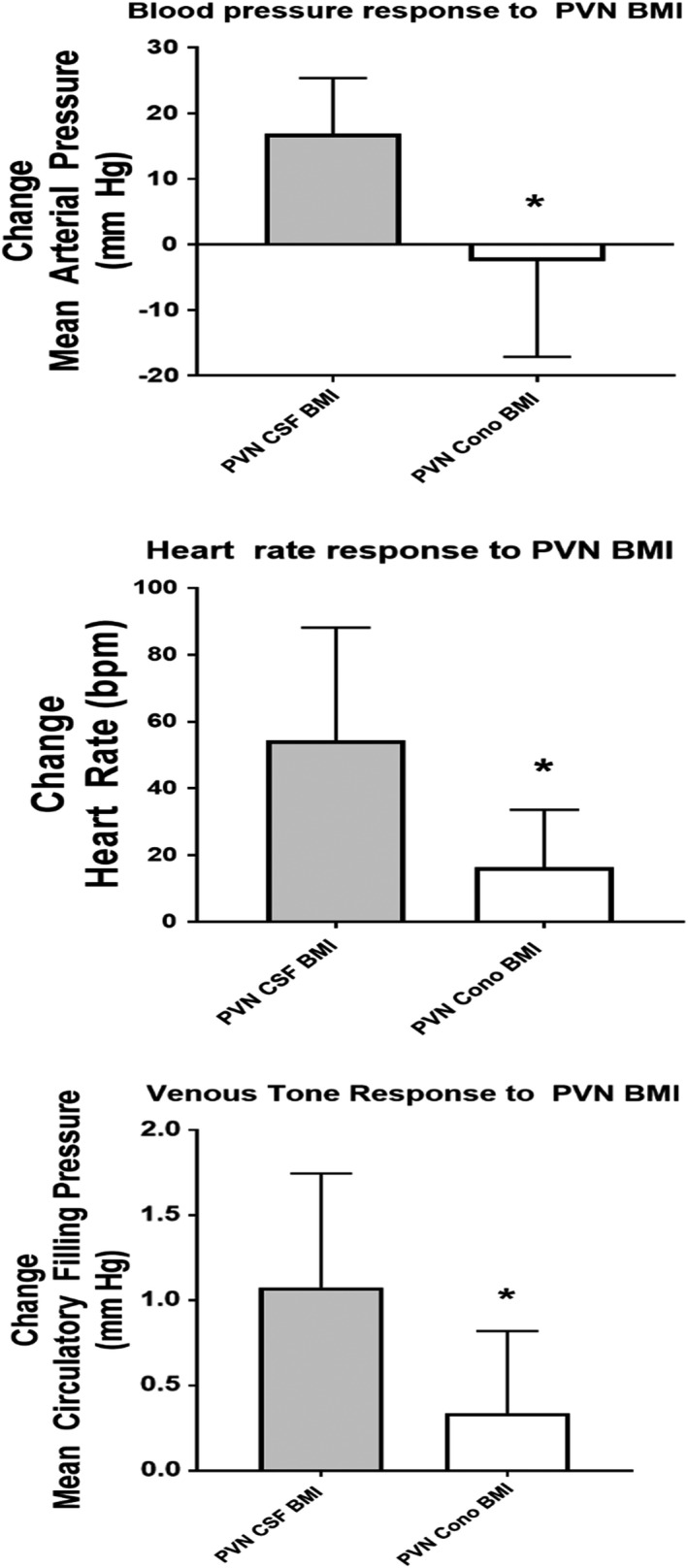
These graphs compare the arterial pressure (MAP), heart rate (HR), and venoconstrictor (MCFP) responses to injection of bicuculline methiodide (BMI) into the PVN after pretreatment of the PVN with vehicle (n = 5) or omega conotoxin GVIA (*n* = 10). ^#^ *p* < 0.05 pre‐ versus post‐PVN conotoxin

As described above, neither injection of vehicle nor omega conotoxin into the PVN region significantly affected baseline values for MAP, HR, or MCFP. In order to serve as both a time and vehicle control, a separate cohort of rats received two injections of vehicle (Veh/Veh) into the PVN region, followed by pericardial BK tested at 10 µg/kg. In this cohort, following the first vehicle injection, pericardial BK increased MAP by 22 ± 8 mm Hg, HR by 31 ± 17 bpm, and MCFP by 1.27 ± 0.38 mm Hg (Figure 2). Responses to pericardial BK following a second injection of vehicle into the PVN region caused hemodynamic changes that were reduced slightly. This reduction reached statistical significance for HR (17 ± 10 bpm). In contrast, pretreatment of the PVN region with omega conotoxin greatly attenuated the responses to pericardial BK injection. After conotoxin injection into the PVN region, pericardial BK caused MAP to increase by −1 ± 3 mm Hg at 1 µg/kg and by 6 ± 3 mm Hg at 10 µg/kg. After conotoxin, pericardial BK caused MCFP to increase by −0.19 ± 0.07 mm Hg at 1 µg/kg and by 0.20 ± 0.18 mm Hg at 10 µg/kg (Figure 2). Similarly, conotoxin treatment attenuated the HR responses to pericardial bradykinin (10 µg/kg: 4 ± 14 bpm). We used injection of bicuculline methiodide (BMI) in order to test the effectiveness of PVN regional conotoxin. Prior to conotoxin, PVN BMI caused increases in MAP 17 ± 9 mm Hg, HR 54 ± 34 bpm, and MCFP of 1.08 ± 0.67 mm Hg, respectively. These responses were attenuated significantly after injection of conotoxin into the PVN region where PVN BMI triggered changes in MAP of −3 ± 15 mm Hg, HR of 16 ± 17 bpm, and in MCFP of 0.34 ± 0.48 mm Hg (Figures 3 and 4).

### 3.4 Histology

**FIGURE 5:**
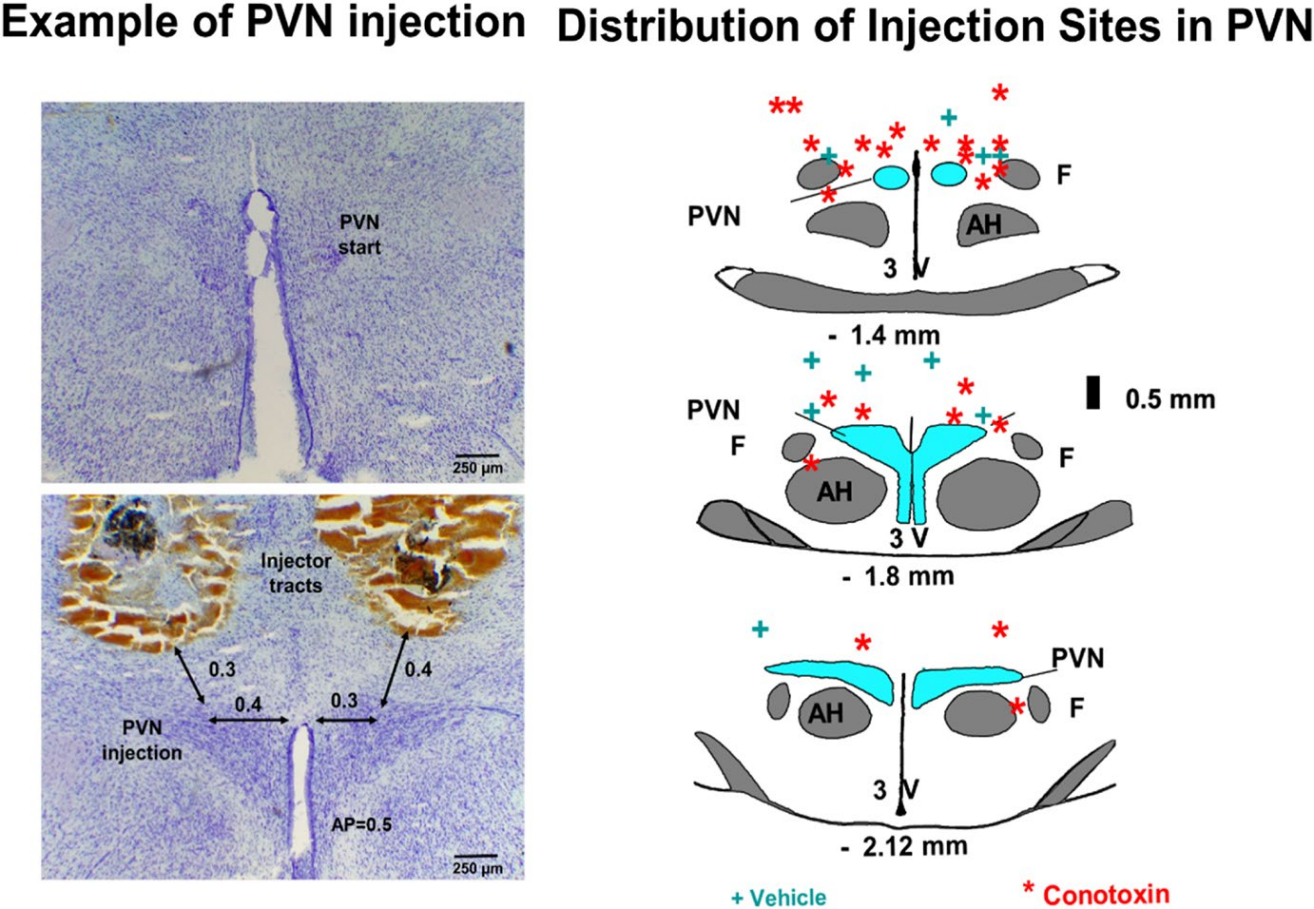
This figure shows a photo of a PVN injection and schematic representations of coronal sections through the rostrocaudal extent of the PVN region. The distance posterior to bregma according to the atlas of Paxinos and Watson is shown at the bottom of each diagram. The site of termination of the injector tracts for vehicle injection is shown by +while those for conotoxin injections are indicated by *. AH; anterior hypothalamus, f; fornix, 3V; third ventricle

The sites of termination of the PVN injectors are shown in Figure 5. On average in rats receiving dual injections of CSF, the injectors terminated approximately 0.16 ± 0.22 mm (right side) and 0.36 ± 0.34 mm (left side) posterior to the beginning of the PVN. In the dorsoventral plane, the injectors on the left side terminated within 0.32 ± 0.28 mm of the PVN, while those on the right side terminated within 0.24 ± 0.47 mm of the PVN. In the rats receiving PVN conotoxin, in the anterior‐posterior plane, injectors were found to be 0.25 ± 0.27 mm posterior to the beginning of the PVN on the left side and 0.38 ± 0.27 mm on the right side. In the dorsoventral plane, the injectors were found at 0.05 ± 0.41 mm on the left side and at 0.13 ± 0.43 mm on the right side. There were no significant differences in these coordinates among the groups.

## 4 DISCUSSION

Veins play an important role in the control of cardiovascular function by regulating venous return, cardiac output, and, therefore, arterial blood pressure. Our previous work showed that pericardial injection of bradykinin increased MCFP, an index of venous tone(Martin et al., 2020). We also reported that disinhibition of the PVN increased venous tone(Martin et al., 2006). In this work, we tested the hypothesis that the PVN region is an essential component of the CSAR arc controlling peripheral venous tone. We observed that injection of the N‐type calcium channel antagonist, omega conotoxin, into the PVN region largely abolished the venous responses evoked by pericardial injection of bradykinin. Thus, neurotransmission within the PVN region is necessary for full expression of the venoconstrictor responses to stimulation of bradykinin‐sensitive pericardial afferents.

Application of bradykinin to the epicardial surface of the heart is recognized as a trigger for the CSAR that, in turn, elicits a strong sympathetically mediated pressor response (Chen et al., 2015; Lu et al., 2017; Veelken et al., 1996; Xu et al., 2013; Zahner & Pan, 2005; Zhang et al., 2012). Since venous function is sensitive to pressures within the chest, we chose to perform our studies in baroreflex intact closed chested rats who had resumed spontaneous breathing. We modeled our approach on that of McDermott et al. (McDermott et al., 1995) who found that intrapericardial injection of bradykinin in awake rats caused dose‐dependent increases in blood pressure with a maximum effect of 25–35 mm Hg. We recently reported similar pressor responses in awake chronically instrumented rats (Martin et al., 2020). The present work was done in closed chested but anesthetized acutely prepared rats where we observed a somewhat reduced pressor responses between 16 and 18 mm Hg. This may represent the effects of anesthesia since other work using anesthetized open chested rat preparations also reported pressor responses ranging between 10 and 25 mm Hg (Lu et al., 2017; Xu et al., 2013; Zahner & Pan, 2005). Additionally, activation of cardiac afferents conveys pain signals to the brain (Fu & Longhurst, 2009; Longhurst, 1984). The anesthetized preparation would lack the perception of pain which may also have attenuated the magnitude of the responses somewhat compared to the conscious state. Nevertheless, our findings in the present study are generally consistent with the established literature for bradykinin‐induced CSAR pressor responses.

Convincing data show that the venous compartment plays an important role in circulatory regulation by controlling venous return(Burgoyne et al., 2014; Fink, 2009; Tyberg, 2002). The CSAR is recognized as a powerful sympathoexcitatory reflex originating from the heart (Chen et al., 2015; Fu & Longhurst, 2009; Longhurst et al., 2001) and sympathetic drive is a major mechanism controlling venous tone (Fink, 2009; Pang, 2001). We hypothesized and were able to show that bradykinin‐induced activation of the CSAR also resulted in significant increases in mean circulatory filling pressure, an accepted index of integrated venous tone (Martin et al., 2020). In the present work, we also observed increases in MCFP during pericardial injection of bradykinin. As with the pressor responses, pericardial bradykinin‐induced increases in venous tone were reduced somewhat compared to our findings in conscious rats. Nevertheless, the increases in mean circulatory filling pressure observed in the present study (~1 mm Hg) would be expected to have a significant hemodynamic effect. It is important to recognize that the venous compartment operates at substantially lower pressures/resistances than the arterial compartment. Consequently, even small changes in venous pressure can have substantial impacts on venous function. The increase in MCFP that we observed represents an increase of approximately 15%–17% of baseline MCFP. Greene showed that a change in systemic filling pressure of 3.4 mm Hg equated to a change in cardiac output of 40% (Greene & Shoukas, 1986). Accordingly, the CSAR‐induced increases in MCFP observed in our study could be predicted to increase cardiac output by 12%–17%. An important caveat to this prediction is that Greene's work was done in dogs. Accordingly, direct extrapolation to the magnitudes of cardiac output responses may not be precise for the rat. Nevertheless, it seems logical that a CSAR‐driven increase in MCFP will lead to an increase in cardiac output. Indeed, CSAR stimulation was reported to increase cardiac output by approximately 14% in the rat (Wang et al., 2017) and we reported that pericardial BK injection increased cardiac output by 12%–20% in male and female rats, respectively (Martin et al., 2022). Thus, activation of bradykinin‐sensitive afferents in the pericardial space induces a venoconstrictor response of sufficient magnitude to influence systemic hemodynamics.

Compared to the arterial circulation, much less is understood regarding the brain sites involved in control of the venous system. The PVN is recognized as an important brain site involved in modulating cardiovascular function (Pyner, 2014, 2021). PVN stimulation is consistently associated with increases in blood pressure, heart rate, and sympathetic outflow (Kenney et al., 2001; Martin & Haywood, 1992; Martin et al., 1991; Zhang et al., 2002). The PVN also receives input from a variety of peripheral sensory afferents(Shenton & Pyner, 2016; Zheng & Patel, 2017). Indeed, several pieces of data indicate that the cardiac sympathetic afferents impinge on the PVN. Activation of the CSAR increased markers of neuronal activation in the PVN (Guo & Moazzami, 2004; Xu et al., 2013), whereas interruption of the PVN via electrolytic lesions (Zhong et al., 2008) or injection of local anesthetics (Xu et al., 2013; Zhong et al., 2008) or a GABA agonist (Xu et al., 2013) attenuated CSAR‐induced blood pressure, heart rate, or renal sympathetic nerve responses. Our work showed that chemical stimulation of the PVN caused venoconstriction via activation of adrenergic nerves (Martin et al., 1997, 2006). Accordingly, it seemed reasonable to hypothesize that the PVN region is also involved in mediating the venous responses to CSAR activation. While seemingly axiomatic, this is nevertheless an interesting question since there is evidence suggesting that the venous and arterial circulations may be controlled independently. Anatomical studies revealed that the sympathetic neurons projecting to arteries and veins were grouped in distinct populations in the mesenteric ganglion (Browning et al., 1999), suggesting the possibility of differential control of these efferent projections. Moreover, functional studies reported different frequency responses in mesenteric arteries and veins (Hottenstein & Kreulen, 1987; Kreulen, 2003), supporting this possibility. Work in humans reported that splanchnic venous capacity was reduced without a change in splanchnic arterial resistance during hemorrhage, providing evidence of differential control of the venous and arterial compartments during reflex activation (Price et al., 1966). Similarly, retrograde tracing studies reported that sympathetic pathways emanating from the PVN are discretely organized (Strack et al., 1989) and that PVN stimulation can produce regionally differentiated sympathetic activation (Kenney et al., 2003). In fact, we observed that PVN stimulation caused a neurally mediated increase in cardiac output with little or no change in total peripheral resistance (Martin & Haywood, 1993) in conscious rats. Moreover, activation of the CSAR was reported to produce regionally differentiated blood flow responses in the renal (decreased) and femoral (increased) circulations (Staszewka‐Barczak et al., 1976). We wished to determine whether the PVN region was involved in CSAR‐induced venous responses by interrupting neurotransmission in the PVN region.

Omega conotoxin GVIA is an N‐type calcium channel antagonist that acts presynaptically to inhibit neurotransmitter release and neurotransmission(Olivera et al., 1994). Injection of omega conotoxin GVIA into the PVN effectively interrupted neurotransmitter release in the PVN and PVN‐mediated behavioral responses (Sanna et al., 2012). Accordingly, we compared responses to pericardial bradykinin injections before and after injection of omega conotoxin GVIA into the PVN region. We tested the effectiveness of this approach by injecting BMI, a GABA‐A antagonist, into the PVN to produce neuronal activation. As we reported previously (Martin et al., 2006), PVN BMI injection was associated with increases in MAP, HR, and MCFP. These responses were largely abolished when the PVN region was pretreated with conotoxin, suggesting relatively effective blockade of neurotransmission in this area.

The observation that injection of conotoxin did not systematically affect baseline blood pressure, heart rate, or MCFP was unexpected. To our knowledge, previous work directly studying the effect of conotoxin injection into the PVN region focused on behavioral effects without hemodynamic assessment. So, there were no previous data on which to base expectations. Early neural recording work showed that autonomic‐related PVN neurons were largely devoid of spontaneous activity(Lovick & Coote, 1988). PVN‐mediated autonomic drive appears to be controlled by an intricate balance of excitatory (e.g. glutamate) and inhibitory (e.g. GABA) inputs (Chen et al., 2003; Pyner, 2014, 2021). In the normal state, these act in a feedback loop to maintain PVN autonomic neurons quiescent due to a dominant influence of GABA (Pyner, 2014, 2021). Therefore, it might be predicted that interruption of GABA release with conotoxin would lead to activation of PVN autonomic drive. However, the cardiovascular response to interruption of GABA function in the PVN also requires background activation of excitatory amino acid receptors (Chen et al., 2003; Li et al., 2006; Mourao et al., 2021) to allow the neuronal membrane potential to approach action potential threshold (Chen et al., 2003; Li et al., 2006). This may involve, at least in part, glutamate release (Li et al., 2006). Thus, by reducing glutamate release, conotoxin may prevent PVN neurons from reaching their firing threshold despite conotoxin‐induced loss of GABA release (Chen et al., 2003; Li et al., 2006; Mourao et al., 2021). Clearly, direct neural recording possibly coupled with microdialysis experiments will be needed to decipher these possibilities.

We also found that pretreatment of the PVN region with conotoxin markedly attenuated the MAP and MCFP responses to pericardial injection of BK. Our findings support prior work indicating that PVN inhibition with muscimol or lidocaine abolished the increases in blood pressure and renal nerve activity caused by pericardial BK(Xu et al., 2013; Zahner & Pan, 2005). Accordingly, we conclude that the PVN region is required for the venoconstrictor responses to activation of bradykinin‐sensitive pericardial afferent that is thought to mediate the CSAR. An important caveat to the present work is the volume of injectate. Based on previous work estimating the zone of distribution of BMI (Segura et al., 1992), we are confident that 100 nl injections of BMI would be contained within the PVN. Since we wished to inhibit neurotransmission in the entire PVN, we chose to use a larger volume of 200 nl for conotoxin injections similar to previous work using conotoxin in the PVN (Sanna et al., 2012). However, we have not measured the zone of distribution of conotoxin. Thus, we cannot rule out the possibility that structures adjacent to the PVN were impacted. Accordingly, our findings are best interpreted as indicating that neurotransmission in and around the PVN region is required CSAR‐induced venoconstriction. Future studies using more discrete approaches (i.e. glass micropipettes and optogenetics) will be necessary to specifically decipher which region or subregion of the PVN area is responsible for these effects.

An additional factor to consider is that the present study was conducted in rats with intact arterial and vagal reflex function. It is conceivable that some degree of attenuation of responses occurred due to reflex compensation. It has been reported that denervation of arterial baroreceptors or cervical vagotomy potentiated the pressor response to activation of the CSAR, while conversely stimulation of vagal afferents attenuated these responses (Gan et al., 2011; Xu et al., 2011). The PVN receives ascending inputs from the nucleus tractus solitarius (NTS) that impinge on presympathetic neurons (Affleck et al., 2012). Moreover, the activity of PVN autonomic‐related neurons is modulated by atrial afferents (Coote, 2005) as well high‐pressure baroreceptors (Dampney et al., 2018; Lovick & Coote, 1988). This opens the possibility that CSAR responses may be modulated by afferent baroreflex input directly at the PVN. However, since cardiac afferent information to the PVN travels through other integration sites such as the NTS and RVLM (Gao et al., 2014), it seems reasonable that the CSAR responses may be modulated at multiple loci in addition to the PVN. These represent interesting questions to be addressed with more defined electrophysiological studies.

The CSAR appears to be activated physiologically via endogenous mediators such as hydrogen peroxide, adenosine, or bradykinin (Chen et al., 2015; Longhurst et al., 2001). It is conceivable that the ongoing release of these substances may contribute to tonic activation of the CSAR, since inhibition of the CSAR with the epicardial application of lidocaine decreased CO (Wang et al., 2017). However, at present, it is not known if inhibition of the CSAR leads to a decrease in venous tone. Increased activity of the CSAR is also linked to number of pathophysiological conditions such as hypertension, heart failure, and coronary ischemia (Chen et al., 2015; Fu & Longhurst, 2009; Li et al., 2013; Wang et al., 2014, 2017; Zhu et al., 2009). These conditions are also associated with an increased in systemic venous tone (Chien et al., 1992; Fink, 2009; Pang, 2001). Teleologically, this response may have evolved as a mechanism to increase or help support (in the case of heart failure) venous return/cardiac output to provide more blood flow to a heart under duress. Interestingly, each of these conditions is also associated with activation of the PVN (Dampney et al., 2018; Koba et al., 2020; Pyner, 2014). The PVN has been linked to control of venous tone (Martin et al., 1992, 1997) and cardiac output (Martin & Haywood, 1993). Thus, the CSAR‐PVN‐venous arc may serve as an important mechanism to regulate venous tone in both physiological and pathophysiological conditions.

There are some limitations to this study. The right atrial balloon inflations were timed at 90–180 s after the pericardial injections as opposed to performing the inflations at the peak of the responses. This was done for practical reasons since it was difficult to anticipate the peak response. There may have been two consequences of this approach. First, in some cases, the balloon inflation may have occurred prior to the peak response, which would have underestimated the MAP, HR, and MCFP responses. Second, this approach may also have introduced additional variability to the measurements given that the time course of the responses could be different among rats. Another potential limitation is the dose of conotoxin. The dosage of conotoxin reported in the literature varies widely. In some cases, as little as 5 ng has been reported to inhibit behavioral effects such as yawning (Succu et al., 1998) to some but not all stimuli. On the other hand, the dose of conotoxin required to inhibit pain responses is substantially higher with doses in the range of 200–300 ng reported to inhibit pain responses in the rat with a duration of up to 2 h (Finn et al., 2003; Scott et al., 2002). Since bradykinin stimulation of pericardial afferents may involve a pain component (Fu & Longhurst, 2009), we chose to use a high dose of conotoxin. This could introduce the potential for effects other than inhibition of presynaptic neurotransmitter release potentially via direct inhibition of presympathetic PVN neurons. Irrespective of the mode of action, however, the data seem clear that injection of conotoxin into this area of the hypothalamus did largely attenuate the venous response to pericardial bradykinin and thus support the main observation that the region in and around the PVN is required for this venoconstrictor response. Lastly, this study was performed using injection of BK into the pericardial space as a stimulus to activate the CSAR. Accordingly, BK likely was circulated throughout the pericardial space triggering activation of all bradykinin‐sensitive pericardial afferents. Thus, the observed responses may be an aggregate response to different types of pericardial afferents. It is possible that pericardial afferent activation with other stimuli (e.g. adenosine and reactive oxygen species) might evoke different responses that may or may not involve venoconstriction. Thus, the current outcomes pertain only to those situations in which cardiac BK is increased. Nevertheless, in conclusion, these findings support our view that the region in and around the PVN is integral to the venoconstriction mediated by BK‐sensitive pericardial afferents.

## CONFLICT OF INTEREST

The authors have no conflicts of interests or competing interests to declare.
